# First person – Klytaimnistra Kiouptsi

**DOI:** 10.1242/bio.040642

**Published:** 2018-11-30

## Abstract

First Person is a series of interviews with the first authors of a selection of papers published in Biology Open, helping early-career researchers promote themselves alongside their papers. Klytaimnistra Kiouptsi is first author on ‘Hypoxia evokes increased PDI and PDIA6 expression in the infarcted myocardium of ex-germ-free and conventionally raised mice’, published in BiO. Klytaimnistra is a postdoc in the lab of Christoph Reinhardt at the Center for Thrombosis and Hemostasis (CTH), University Medical Center Mainz, Germany, investigating gut microbiota and its role in cardiovascular disease.


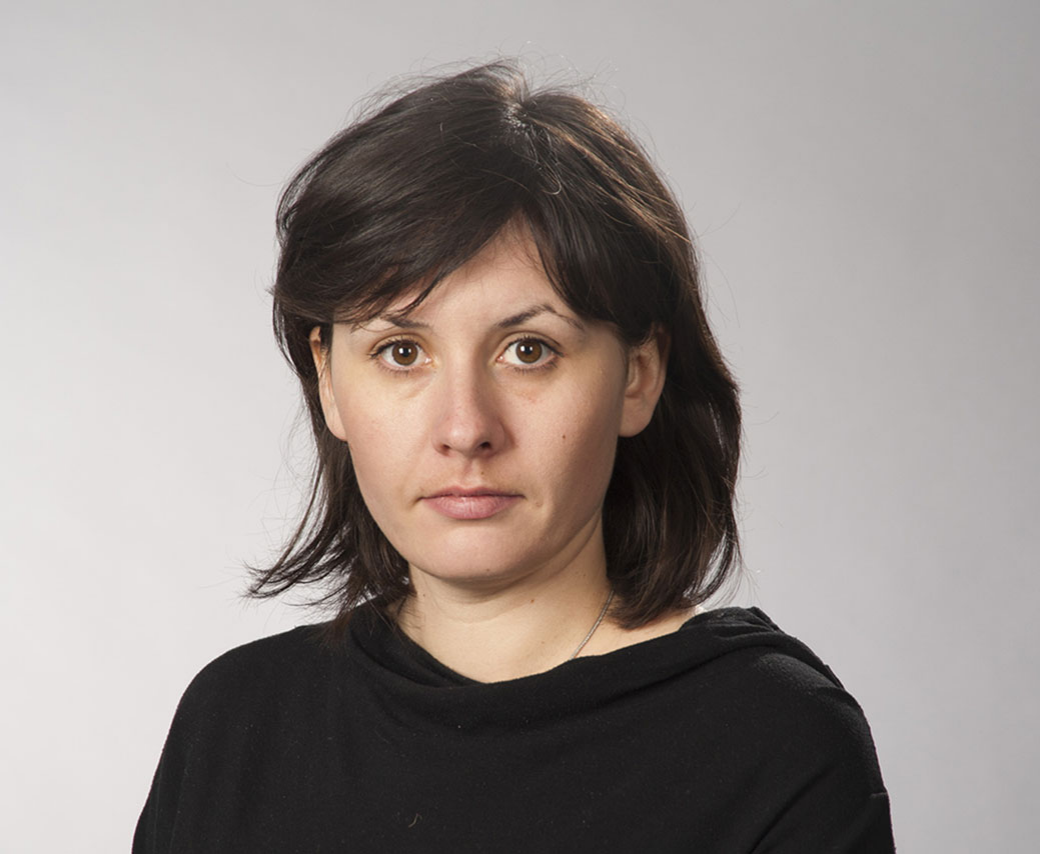


**Klytaimnistra Kiouptsi**

**What is your scientific background and the general focus of your lab?**

I hold a Master's degree in Biotechnology from the University of Ioannina in Greece. My interest in thrombosis research and platelet function led me to enrol in the PhD programme at the Mainz Research School of Translational Biomedicine. During my PhD in the lab of Christoph Reinhardt at the Center for Thrombosis and Hemostasis in Mainz, I investigated the role of the gut microbiota in promoting arterial thrombosis and its implication in myocardial infarction. More specifically, I studied the hypoxia-dependent regulation of protein disulphide isomerase expression. That was when I first started learning about the gut microbiome and the ability of microbes to influence cardiovascular functions and thrombus growth. Prof. Reinhardt uses his gnotobiotic facility to explore the influence of gut microbes in thrombosis and cardiovascular disease.

“[…] the influence of the gut microbiota is not only restricted to the intestine but also affects other organs’ functions.”

**How would you explain the main findings of your paper to non-scientific family and friends?**

During myocardial infarction, which is commonly known as a heart attack, there is decreased blood flow to one part of the heart, resulting in low oxygen and nutrient supply, which causes damage to the heart muscle. However, there are certain molecules at the site of infarction, which protect the heart from going into hypoxic stress. In our study, we identified one of these factors, namely PDIA6. We revealed that the influence of the gut microbiota is not only restricted to the intestine but also affects other organs’ functions. Using germ-free mouse models, our research showed that the microbiota contributes to increased PDIA6 expression in the infarcted mouse heart.

**What are the potential implications of these results for your field of research?**

Based on our results, it will be interesting to explore how the colonization with a gut microbiota, a chronic inflammatory stimulus, affects platelet function and the development of cardiovascular disease. So far, relatively little is known on the influence that this microbial ecosystem exerts on vascular phenotypes.

**What has surprised you the most while conducting your research?**

It is most fascinating to me to realize that the gut microbiota has remote effects, leading to adaptive changes in distant organs. Our laboratory was able to demonstrate that platelet deposition in arterial thrombosis and cardiovascular functions are influenced by the gut microbiota.

**What, in your opinion, are some of the greatest achievements in your field and how has this influenced your research?**

Gnotobiotic isolator technology is a powerful tool to identify host-microbial interactions and to pinpoint the mechanisms that colonizing gut microbes influence in the host. Combined with genetic models and with established methods to study organ functions, gnotobiotics will advance new insights in the area of vascular physiology.

**What changes do you think could improve the professional lives of early-career scientists?**

Major problems in academia are the restricted number of grants, short-term contracts for researchers and low remuneration, in combination with a very challenging time schedule. These obstacles often urge highly educated and bright people to seek their career path in a different field. A solution would be to increase funding to provide long-term perspectives to successful scientists. This will not only improve the quality of publications, but will also lead to basic research that is of high originality.

**What's next for you?**

At this point, I am on maternity leave as my second child was born at the end of October. I am looking forward to returning to the lab once my daughter is older and has a spot in day care.

**Figure:**
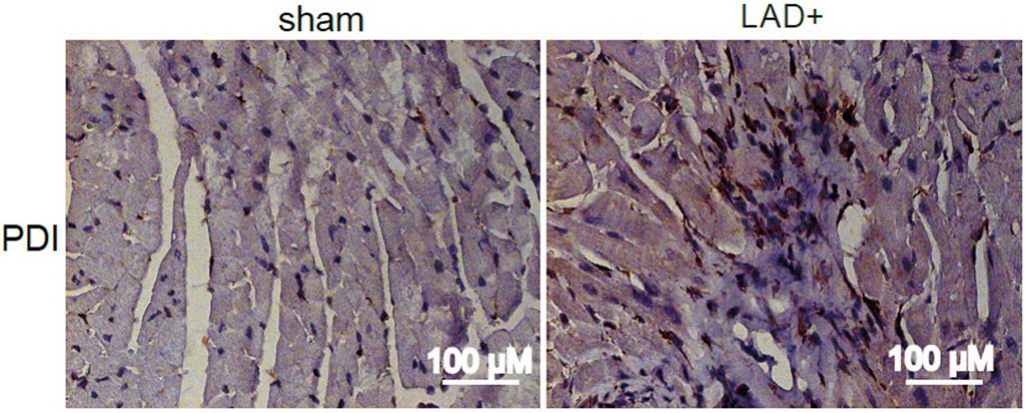
**Immunohistochemistry staining for protein disulphide isomerase (PDI) in sham-operated (left) and left anterior descending artery (LAD)-ligated (right) heart tissue.**
